# Thermally Driven Structural Order of Oligo(Ethylene Glycol)-Terminated Alkanethiol Monolayers on Au(111) Prepared by Vapor Deposition

**DOI:** 10.3390/molecules27175377

**Published:** 2022-08-23

**Authors:** Young Ji Son, Hungu Kang, Sicheon Seong, Seulki Han, Nam-Suk Lee, Jaegeun Noh

**Affiliations:** 1 Department of Chemistry, Hanyang University, 222 Wangsimni-ro, Seongdong-gu, Seoul 04763, Korea; 2 Department of Chemistry, Korea University, 145 Anam-ro, Seongbuk-gu, Seoul 02841, Korea; 3 National Institute for Nanomaterials Technology, Pohang University of Science and Technology, Pohang 37673, Korea; 4 Institute of Nano Science and Technology, Hanyang University, 222 Wangsimni-ro, Seongdong-gu, Seoul 04763, Korea

**Keywords:** oligo(ethylene glycol)-terminated alkanethiols, self-assembled monolayers, surface structure, electrochemical behavior, temperature effect, scanning tunneling microscopy

## Abstract

To probe the effects of deposition temperature on the formation and structural order of self-assembled monolayers (SAMs) on Au(111) prepared by vapor deposition of 2-(2-methoxyethoxy)ethanethiol (CH_3_O(CH_2_)_2_O(CH_2_)_2_SH, EG2) for 24 h, we examined the surface structure and electrochemical behavior of the resulting EG2 SAMs using scanning tunneling microscopy (STM) and cyclic voltammetry (CV). STM observations clearly revealed that EG2 SAMs vapor-deposited on Au(111) at 298 K were composed of a disordered phase on the entire Au surface, whereas those formed at 323 K showed improved structural order, showing a mixed phase of ordered and disordered phases. Moreover, at 348 K, uniform and highly ordered EG2 SAMs on Au(111) were formed with a (2 × 3√3) packing structure. CV measurements showed sharp reductive desorption (RD) peaks at −0.818, −0.861, and −0.880 V for EG2 SAM-modified Au electrodes formed at 298, 323, and 348 K, respectively. More negative potential shifts of RD peaks with increasing deposition temperature are attributed to an increase in van der Waals interactions between EG2 molecular backbones resulting from the improved structural quality of EG2 SAMs. Our results obtained herein provide new insights into the formation and thermally driven structural order of oligo(ethylene glycol)-terminated SAMs vapor-deposited on Au(111).

## 1. Introduction

Closely packed and highly ordered self-assembled monolayers (SAMs) can be prepared by the spontaneous adsorption on metals of organic molecules containing chemically active sulfur, selenium, carboxylic or alkyne anchoring groups [1,2,3,4,5,6,7,8,9,10,11,12,13,14,15,16,17,18,19,20]. As a result, SAMs provide a very useful route for the fabrication of multifunctional molecular thin films that can be applied to various practical applications in the fields of biotechnology and nanotechnology [21,22,23,24,25,26,27,28,29]. In particular, oligo(ethylene glycol) (OEG)-terminated alkanethiol SAMs on metals have drawn much attention due to their effective inhibition of nonspecific protein adsorption [30,31,32,33]. It has been found that the orientation, structural order, conformation, and internal and terminal hydrophilicity of OEG SAMs significantly affect the interfacial properties between the SAM surface and proteins, thereby defining their nonspecific adsorption characteristics [30,31,32,33]. It was revealed that highly ordered, crystalline, OEG-based SAMs on Ag surface showed low ability to resist protein adsorption [32]. However, such crystalline structures are much more interesting for molecular electronics considering surprisingly high conductance for this type of molecular backbone [34]. Therefore, understanding the surface structures and conformations of OEG SAMs is essential. 

Spectroscopic and X-ray adsorption measurements show that OEG SAMs have three different conformations: all-trans, helical, or amorphous. The conformation largely depends on the surface coverage, the number of ethylene glycol units (EG)_n_, the type of metal, the properties of the solvent, and the deposition method [32,33,35,36,37]. It has been reported that on an Au surface, OEG SAMs with an (EG)_4_ unit have mixed conformations containing helical and all-trans structures in the EG groups [32,35]. It has also been found that when the number of EG units is sufficiently high (*n* ≥ 6), OEG SAMs prefer to have helical conformations on the Au surface. Atomic force microscopy (AFM) measurements showed the first molecular scale features of OH-(EG)_6_-terminated alkanethiol SAMs on Au(111), indicating a closely packed (√3 × √3)30° structure. Interestingly, nanometer-scale defects with a depth of ~2 nm were often observed in the ordered domains, which are not observed in OH-terminated alkanethiolate SAMs [38]. High-resolution scanning tunneling microscopy (STM) measurements also elucidated that CH_3_-(EG)_2_-terminated alkanethiol SAMs deposited from a 1 mM ethanol solution on Au(111) for 24 h had very unique structural features: a disordered phase (amorphous phase) and an ordered phase with a (√3 × 7) structure [39].

It has been demonstrated that the domain formation and structural order of organic SAMs on metals are considerably influenced by SAM preparation conditions, such as deposition time, deposition temperature, deposition method (solution vs. vapor), and solvent properties [4,7,24,39,40,41,42,43,44,45,46,47,48,49]. Molecular-scale STM studies have shown that well-ordered organic thiol or selenol SAMs on Au(111) with large ordered domains and few structural defects are formed at high deposition temperatures [4,7,42,43,44,45]. It has been suggested that the markedly improved structural quality of organic SAMs at high deposition temperature is due to a lowering of the diffusion barrier of adsorbates during adsorption [50]. STM observations clearly reveal that ambient-pressure vapor deposition is a very simple and useful technique that remarkably enhances the structural order of SAMs on Au(111) derived from alkanethiols, alkyl thiocyanates, and alkyl selenocyanates. This enhancement is due to eliminating possible negative effects that can be induced by solvent-adsorbate interactions during self-assembly in solution [7,46,51,52]. In spite of numerous studies regarding the effect of deposition temperature on the formation and structure of alkanethiol and alkaneselenol SAMs [1,4,7,42,43,44,45], there are no molecular-scale STM studies to date regarding temperature-dependent OEG SAM formation on Au(111) by ambient-pressure vapor deposition. 

To understand these issues clearly, we examine the surface structures and electrochemical behaviors of OEG SAMs on Au(111) prepared by vapor deposition of 2-(2-methoxyethoxy)ethanethiol [CH_3_O(CH_2_)_2_O(CH_2_)_2_SH, CH_3_(EG)_2_SH] by using STM and cyclic voltammetry (CV). In this study, we report the first STM results showing the structural transitions of CH_3_(EG)_2_S SAMs on Au(111) with increasing vapor deposition temperature from a disordered phase to an ordered phase with a (2 × 3√3) rect structure via an intermediate phase containing ordered and disordered phases. 

## 2. Experimental

### 2.1. Chemicals and Preparation of Au(111) Substrates

CH_3_(EG)_2_SH were purchased from Sigma-Aldrich (Munich, Germany). Freshly cleaved mica sheets were preheated at 623 K in the vacuum chamber. Subsequently, Au with a thickness of 100 nm was deposited onto the mica surface under a vacuum pressure of ~10^−5^ to 10^−6^ Pa. Deposition rate of gold was 2–3 Ås^−1^. The deposited Au films on mica were annealed at 623 K in the same vacuum chamber for 2 h. This procedure results in the fabrication of Au substrates consisting of large, atomically flat terraces in the size range of 100–400 nm and exhibiting a (111) single crystal facet. 

### 2.2. Preparation of CH_3_(EG)_2_S SAMs

**Figure 1:**
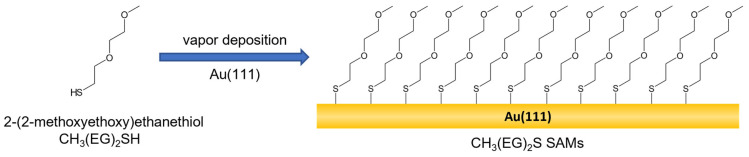
Chemical structure of CH_3_(EG)_2_SH and formation of CH_3_(EG)_2_S SAMs on Au(111) via vapor deposition.

CH_3_(EG)_2_S (simply, EG2) SAMs were prepared by the ambient-pressure vapor deposition method by placing the Au(111) substrates into a 3 mL V-vial containing a 1 μL neat liquid of CH_3_(EG)_2_SH (Figure 1). The vials were then tightly sealed with a cap and kept at the desirable deposition temperatures of 298, 323, or 348 K for 24 h. The prepared EG2 SAMs on Au(111) were thoroughly rinsed with plenty of pure ethanol to remove any contaminants from the SAM surfaces and were dried by N_2_ gas before surface characterization.

### 2.3. STM and CV Measurements

STM observations were performed using a NanoScope E (Veeco, Instruments Inc., Santa Barbara, CA, USA). The tips were fabricated mechanically by cutting Pt/Ir (80:20) wire with diameter of 0.25 mm. All STM data were collected in constant current mode using bias voltages between 300 mV and 500 mV and tunneling currents between 200 pA and 500 pA. CV measurements were carried out using an electrochemical analyzer (BAS-100; Bio Analytical Systems, West Lafayette, IN, USA). A typical three-electrode cell was composed of an EG2 SAM-covered Au working electrode, a Pt wire counter electrode, and an Ag/AgCl glass reference electrode. The reductive desorption CVs for EG2 SAMs on Au(111) electrodes were recorded in a N_2_-bubble-treated 0.1 M KOH electrolyte solution by cycling in the potential range of 0 to −1.2 V at a rate of 0.4 Vs^−1^.

## 3. Results and Discussion

### 3.1. Deposition Temperature-Dependent Structural Transitions of EG2 SAMs on Au(111) Formed by Vapor Deposition 

**Figure 2:**
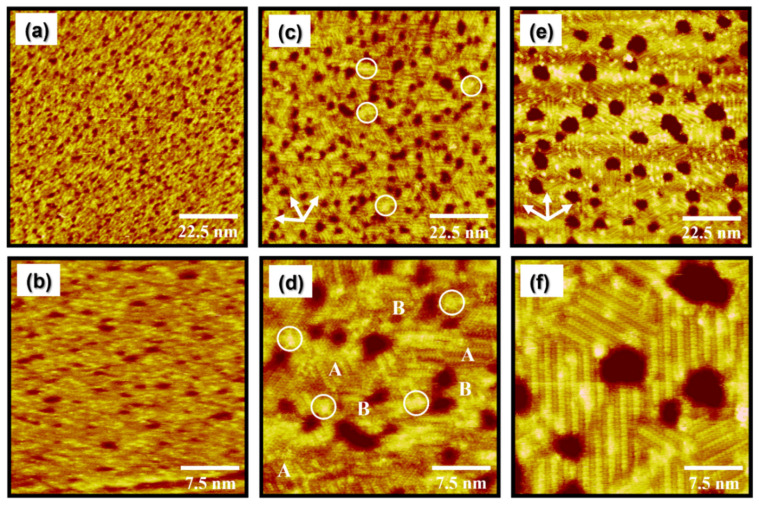
STM images of EG2 SAMs on Au(111) formed over 24 h at a vapor deposition temperature of (**a**,**b**) 298 K, (**c**,**d**) 323 K, and (**e**,**f**) 348 K. Scan sizes of STM images are 90 × 90 nm^2^ (**a**,**c**,**e**) and 30 × 30 nm^2^ (**b**,**d**,**f**). Note that A and B in Figure 2d correspond to the ordered domains and the disordered domains, respectively.

OEG SAMs formed by solution deposition on gold surfaces have been extensively studied for understanding the surface structure and adsorption geometry of OEG SAMs because these structural characteristics markedly affect the adsorption behavior of biomolecules [29,30,31,32,33,35,36,37,38,39]. The first molecular-scale STM study showed that EG2 SAMs with a small number of EG units deposited via solution on Au(111) had inhomogeneous surface structures, showing the coexistence of well-ordered molecules and poorly ordered molecules with a bright contrast in the SAMs, unlike alkanethiol SAMs. Therefore, it was proposed that the formation of this unique disordered phase is ascribed to the electrostatic repulsions between the EG chains during SAM formation [39]. In contrast, vapor deposition at an elevated temperature can be very effective for improving the structural quality of SAMs compared to solution deposition [7,46,51,52]. In this regard, we used STM and CV to characterize EG2 SAMs vapor-deposited on Au(111) for 24 h as a function of deposition temperature. STM observations clearly show that the surface morphology of EG2 SAMs on Au(111) were remarkably dependent on deposition temperature, as shown in Figure 2. The STM images of Figure 2a,b show that EG2 SAMs vapor-deposited on Au(111) at 298 K for 24 h have a fully disordered phase with many vacancy islands (VIs, dark defects). Such VIs, with a monatomic height of the Au lattice, could be formed through chemisorption of organic molecules containing an active anchoring group, such as sulfur or selenium, onto the gold surface, as reported in many previous papers [1,2,3,4,5,7,38,39,40,41,42,43,44,45,46,47,48,49,50,51,52]. Therefore, the presence of VIs in EG2 SAMs on Au(111) implies that chemisorbed EG2 monolayers were formed by vapor deposition, as is also the case with EG2 SAMs formed by solution deposition [39]. Interestingly, a partially ordered phase of EG2 SAMs was formed after 24 h deposition in a 1 mM ethanolic solution at 298 K [39], whereas a fully disordered phase was formed via vapor deposition using the same deposition time and temperature. This result strongly suggests that ethanol (polar protic solvent) promotes the formation of ordered EG2 monolayers to some extent by stabilizing the monolayers through hydrogen bonding interactions between the solvent and the EG2 molecules during SAM formation. This suggestion is supported by the fact that the disordered phase of EG2 SAMs was solely formed from hexane (nonpolar solvent) (data not shown here). It has been reported that solvent properties can markedly affect the domain formation and structural order of thiolate SAMs on Au(111) [47,53,54,55].

It has been demonstrated that higher thermal energy can greatly increase the diffusion rate of adsorbates and/or adsorbate–metal complexes during SAM formation, resulting in the formation of organic SAMs with improved structural quality showing large ordered domains and few structural defects [1,4,7,42,43,44,45]. When the vapor deposition temperature increases from 298 to 323 K, the surface features of the EG2 SAMs on Au(111) remarkably changed from the disordered phase to a mixed phase of ordered and disordered phases with many small bright aggregates (the white circle) on the entire Au(111) surface, as shown in Figure 2c,d. It is clear from the STM image that the ordered phase was the dominant phase formed. In contrast, solution-deposited EG2 SAMs at the same preparation conditions (deposition temperature of 323 K and deposition time of 24 h) were dominated by the disordered phase, suggesting that solvent molecules with high thermal energy significantly prevent two-dimensional (2D) ordering of EG2 SAMs at high solution temperature [56]. Our STM results strongly suggest that the structural order of EG2 SAMs was remarkably enhanced by vapor deposition and high deposition temperatures. On the other hand, it was found that the size of ordered domains in vapor-deposited EG2 SAMs ranged from approximately 2 to 8 nm, which were much smaller than those formed in solution-deposited EG2 SAMs, with the ordered domains sized larger than 60 nm formed even at a lower temperature of 298 K. From this result, we also consider that ethanol solvent considerably contributes to the formation of large ordered single domains even though poorly ordered molecular aggregates were embedded in the ordered domains [39]. Tri-directional small ordered domains rotated by a 60° or 120° angle are clearly visualized in Figure 2c (indicated by the arrows), suggesting that the formation of EG2 SAMs is due to the chemical interactions between the sulfur anchoring group and the Au(111) surface, as in the case of long-chain alkanethiol SAMs [5,35,36,37,38,39,40]. The magnified STM image in Figure 2d shows ordered molecular rows (indicated by A) and randomly adsorbed disordered regions (indicated by B). This STM observation revealed that although the structural order of EG2 SAMs is drastically enhanced at the deposition temperature of 323 K (Figure 2c,d), the uniform and fully ordered EG monolayers are still not formed. 

When the deposition temperature was increased from 323 K to 348 K, the surface structures of EG2 SAMs on Au(111) completely changed from a mixed phase to a fully ordered phase, as shown in Figure 2e,f. The magnified STM image in Figure 2f clearly shows that the disordered phases evident at 298 K or 323 K completely disappeared at 348 K. Moreover, the size of the ordered domains drastically increases from a few nanometers to larger than 10 nm at this elevated temperature. Interestingly, many small bright spots in the ordered domains conspicuously appeared, which have never been observed in alkanethiol SAMs [41,42,43,44,45]. It was found that a helical conformation of OEG SAMs is dominantly formed when the number of (EG)_n_ units, n, is larger than 5, whereas OEG SAMs with the (EG)_4_ unit have a mixed conformation of helical and all-trans structures [32]. Based on this conformational change of EG units from the helical conformation to all-trans conformation as the number of EG units decreases, we assume that EG2 SAMs with two EG units prefer to have an all-trans conformation. Therefore, it is reasonable to consider that the well-ordered domains of EG2 SAMs on Au(111) have an all-trans conformation. From a molecular-scale STM study, we revealed that the uniform and well-ordered EG2 SAMs were formed by vapor deposition at a high deposition temperature of 348 K for 24 h. In addition, the phase transitions of EG2 SAMs on Au(111) occur from a disordered phase to a fully ordered phase via an intermediate phase showing the coexistence of two phases, i.e., the ordered and disordered phases. 

### 3.2. Deposition Temperature-Dependent Changes to the Number and Size of VIs in EG2 SAMs on Au(111) Formed by Vapor Deposition 

**Figure 3:**
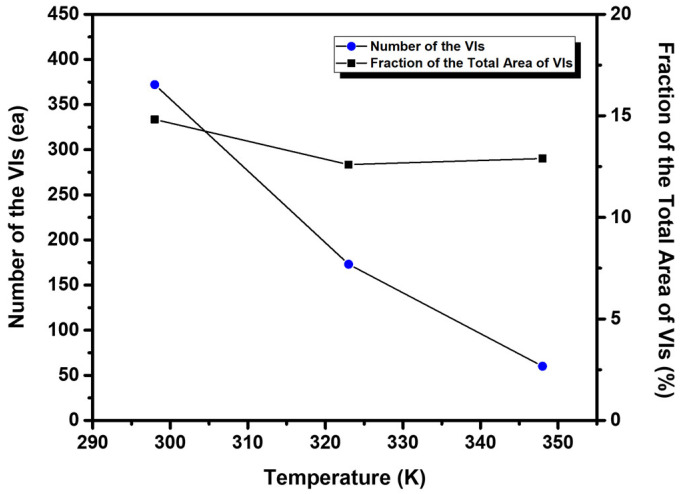
The number of VIs (circles) and the fraction of total area of the VIs (squares) for EG2 SAMs on Au(111) formed at 298 K, 323 K, and 348 K.

As the vapor deposition temperature was increased, another remarkable structural change was observed in EG2 SAMs on Au(111) in the number and size of VIs in the SAMs (Figure 2). Figure 3 shows the number of VIs (circles) and the proportion of the total area of the VIs relative to the total surface area (squares), calculated from several hundred square STM images of SAMs formed at different deposition temperatures. The number of VIs becomes smaller, and the size of VIs becomes larger when the deposition temperature is higher. This trend is a signature of the thermodynamically driven Ostwald ripening process, which entails the growth of the more energetically favorable large VIs at the expense of small VIs in the SAMs, as proposed in previous work [42,50,57,58]. It was also found that the fraction of area of the VIs on the surface was 13–15%, which varied minimally with deposition temperature. The observed temperature-dependent growth of VIs in EG2 SAMs is quite similar to that of decanethiol SAMs [42]. This result implies that the formation and growth of VIs is directly related to the formation of the chemical S-Au bond between the thiol anchoring group and the Au surface, not due to the strength of van der Waals interactions between molecular backbones, as suggested by previous work [59]. 

### 3.3. Packing Structure of Well-Ordered EG2 SAMs on Au(111) Formed by Vapor Deposition at 348 K

**Figure 4:**
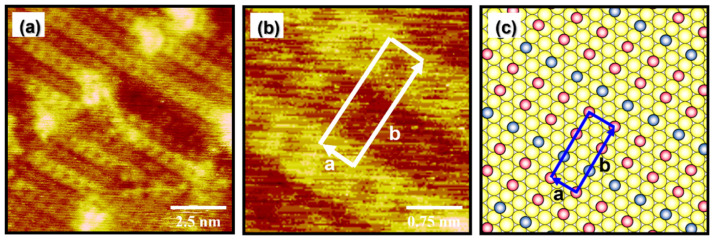
(**a**,**b**) Molecularly resolved STM images of EG2 SAMs on Au(111) formed at 348 K for 24 h via vapor deposition. (**c**) A proposed structural model of EG2 SAMs on Au(111). Scan sizes of STM images are 10 × 10 nm^2^ (**a**) and 3 × 3 nm^2^ (**b**).

High-resolution STM images in Figure 4a,b show that the EG2 SAMs on Au(111) prepared by vapor deposition at 348 K for 24 h exhibit a well-ordered packing structure. Based on these STM images, the lattice parameters of a rectangular unit cell were extracted: a = 5.8 ± 0.2 Å = 2a_h_ and b = 15.0 ± 0.2 Å = 3√3a_h_. Note that the a_h_ is 2.89 Å, corresponding to the distance between gold atoms in the Au(111) lattice. Figure 4c shows a proposed structural model of the well-ordered phase of EG2 SAMs on Au(111), which is described as a (2 × 3√3) structure. The observed structure is comparable to solution-deposited EG2 SAMs with a (√3 × 7) structure formed at 298 K for 24 h [39]. The unit cell contains three adsorbed molecules, and the average area per molecule for the EG2 SAMs was calculated to be 29.0 Å^2^/molecule, which is slightly larger than that of EG2 SAMs solution-deposited at 298 K for 24 h that have a (√3 × 7) structure (25.3 Å^2^/molecule). Therefore, vapor-deposited EG2 SAMs are a bit more loosely packed than solution-deposited SAMs. However, it was found that vapor-deposited EG SAMs at 348 K have much more uniform and well-ordered SAMs compared to solution-deposited EG2 SAMs that contain large areas of disordered phase when formed at 298 K or 323 K [39,56]. 

### 3.4. Reductive Desorption (RD) Behaviors of EG2 SAMs on Au(111) Prepared by Vapor Deposition 

**Figure 5:**
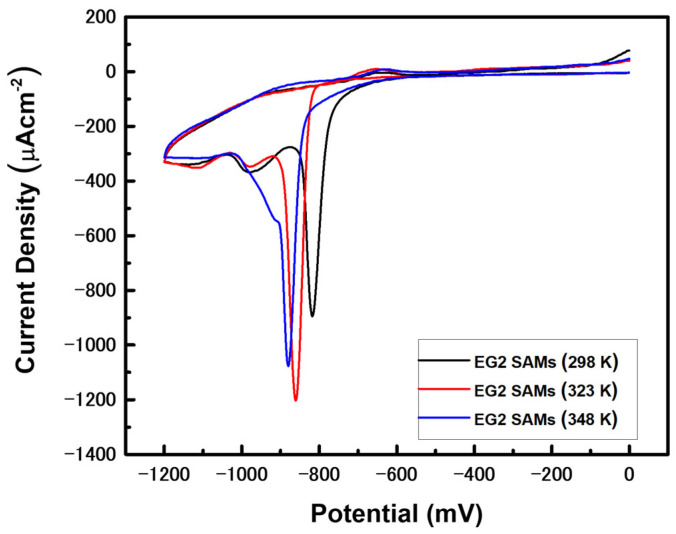
Cyclic voltammograms (CVs) of the reductive desorption of EG2 SAM-modified Au working electrodes formed at the various deposition temperature of 298 K, 323 K, and 348 K in a 0.1 M KOH solution. The reductive desorption CVs were recorded by cycling in the potential range of 0 to −1.2 V at a rate of 0.4 Vs^−1^.

The position and shape of RD peaks for SAM-modified Au electrodes give valuable information regarding the binding affinity between adsorbates and metals, van der Waals interactions between molecular backbones of adsorbed molecules, SAM surface coverage, and the structural quality of the monolayers [46,60,61,62,63]. To probe the electrochemical behaviors of EG2 SAMs prepared at different deposition temperatures on Au(111), we measured the CVs of RD of EG2 SAM-modified Au electrodes prepared by vapor deposition, as shown in Figure 5. The sharp RD peaks for EG2 SAM-modified Au electrodes formed at 298, 323, and 348 K were observed at −0.818, −0.861, and −0.880 V, respectively. It was found that the RD peaks shifted to more negative potentials when the vapor deposition temperature increased. Therefore, we consider that the shift of RD peaks to more negative potential is related to the structural quality of EG2 SAMs on Au(111), since the structural order of EG2 SAMs is greatly improved with increasing deposition temperature (Figure 2). It is well known that the strong van der Waals interactions between molecular backbones is a dominant factor for the formation of 2D-ordered SAMs, resulting in a shift of RD potential to more negative values [46,61,62,63]. Similar RD potential shifts were observed for dodecanethiol SAMs; the sharp RD peak for well-ordered dodecanethiol SAMs appears at −1.028 V, whereas two broad RD peaks for poorly ordered monolayers appear at the less negative potentials of −0.671 and −0.946 V [46]. Moreover, the RD peaks for well-ordered alkanethiol SAMs increases with increasing alkyl chain length due to an increase in van der Waals interactions [63]. From the CV measurements, we clearly demonstrate that the RD peaks of EG2 SAMs shift to more negative potential with increased deposition temperature, which is attributed to an increase in van der Waals interactions between EG2 molecular backbones resulting from the improved structural quality of EG2 SAMs. 

## 4. Conclusions

Surface structures and electrochemical behaviors of EG2 SAMs on Au(111) prepared by vapor deposition for 24 h were investigated by STM and CV to understand the effect of deposition temperature on the formation and structural order of EG2 monolayers. STM observations clearly revealed that EG2 SAMs vapor-deposited on Au(111) at 298 K consisted of a fully disordered phase, whereas those formed at 323 K showed considerably improved structural order with a mixed phase of ordered and disordered phases. When deposition temperature increases to 348 K, the surface structures of EG2 SAMs on Au(111) completely changed from a mixed phase to a fully ordered phase, which can be described as a (2 × 3√3) packing structure. Moreover, as the deposition temperature increased, the number of VIs decreases, and the size of VIs increases, which is thermodynamically driven by the Ostwald ripening process. CV measurements showed sharp RD peaks at −0.818, −0.861, and −0.880 V for EG2 SAM-modified Au electrodes formed at 298, 323, and 348 K, respectively. These shifts of RD peaks to more negative potential are related to the structural quality of the EG2 SAMs on Au(111) and show that the structural order of EG2 SAMs was greatly improved with increasing deposition temperature. In this study, we clearly demonstrate that uniform and highly ordered EG2 SAMs on Au(111) with a high electrochemical stability could be fabricated by vapor deposition at a high deposition temperature of 348 K for 24 h. 

## Data Availability

The data presented in this study are available in this article.

## Sample Availability

SAM samples deposited on Au(111) are available from the authors.
